# Soy Protein Alleviates Malnutrition in Weaning Rats by Regulating Gut Microbiota Composition and Serum Metabolites

**DOI:** 10.3389/fnut.2021.774203

**Published:** 2021-11-29

**Authors:** Zuchen Wei, Nong Zhou, Liang Zou, Zhenxing Shi, Baoqing Dun, Guixing Ren, Yang Yao

**Affiliations:** ^1^Institute of Crop Science, Chinese Academy of Agricultural Sciences (CAAS), Beijing, China; ^2^Laboratory for Green Cultivation and Deep Processing of Three Gorges Reservoir Area's Medicinal Herbs, College of Life Science and Engineering, The Chongqing Engineering, Chongqing Three Gorges University, Chongqing, China; ^3^Key Laboratory of Coarse Cereal Processing, Ministry of Agriculture and Rural Affairs, Chengdu University, Chengdu, China

**Keywords:** untargeted metabolomics, undernourished, microbiota, 16S rRNA, soy protein

## Abstract

Dietary intervention with plant protein is one of the main methods that is used to lessen the symptoms of malnutrition. Supplementary soy protein to undernourished weaning rats for 6 weeks significantly increased their body weight gain. After the intervention, the level of total short-chain fatty acids (SCFAs) was restored to 1,512.7 μg/g, while the level was only 637.1 μg/g in the 7% protein group. The amino acids (valine, isoleucine, phenylalanine, and tryptophan) increased in the colon, and vitamin B_6_ metabolism was significantly influenced in undernourished rats. The tryptophan and glycine-serine-threonine pathways were elevated, leading to an increase in the level of tryptophan and 5-hydroxytryptophan (5-HTP) in the serum. In addition, the relative abundance of *Lachnospiraceae*_NK4A136_group and *Lactobacillus* increased, while *Enterococcus* and *Streptococcus* decreased compared to undernourished rats. Overall, soy protein improved the growth of rats with malnutrition in early life by regulating gut microbiota and metabolites in the colon and serum.

## Introduction

In 2018, stunting affected an estimated 21.9% of children under five (or 149 million), worldwide (1). Emerging views are of the opinion that an immature intestinal microbiota may cause severe malnutrition in children (2). The structure of the gut bacteria and their relative abundance in a healthy host are fixed with beneficial bacteria, such as *Lactobacilli* and *Bifidobacterium*. However, the structure of gut microbiota was vulnerable to the change of internal and external environment. Recently, some researchers demonstrated that bacteria in younger healthy children dominated their malnutrition, which suggests that the growth of the intestinal flora did not keep up with the development of the body (3). Bacteria, such as *Streptococcus* and *Enterococcus* dominated in the intestines of undernourished children and were defined as signs of immature intestines (4). Proteins were utilized by mature healthy intestinal microbes and produced short-chain fatty acids (SCFAs) to regulate tryptophan and 5-hydroxytryptamine (5-HT) in serum. These metabolites regulate host metabolism, appetite, and mood (5). However, there are limited studies on the correlation between serum metabolites and malnutrition.

Known as many reasons for malnutrition, diet is more effective than other factors in regulating the gut microbiota; thus, high-quality food is considered as one of the most effective ways for the treatment of malnutrition (6). Casein has been proven to carry innumerable nutritional values for humans, and it has long been used in the diets of infants to provide nutritional support (7). Casein can easily degrade in the small intestine, and is subsequently available for bacteria in the large intestine to increase the communities of *Lactobacilli* and *Bifidobacterium* while decreasing the counts of *Staphylococci, Coliforms*, and *Streptococci* (8–12). However, casein is not easily available to children in the undeveloped areas, especially in the remote areas (13, 14). Renewed interest mainly focuses on plant-derived protein, especially on soy protein, which is considered as an alternative nutrition for casein. In addition, accumulating studies demonstrated that the absorption of soy protein is more efficient in improving malnutrition than that of the consumption of the mixture of amino acids. In addition, soy protein has also been claimed to play positive roles in modulating the beneficial bacterial composition in the gut with increased communities of *Escherichia* and *Propionibacterium* (15, 16). It has been reported that food supplemented with soy protein alleviated malnutrition by changing the ratio of beneficial and “age-discriminatory” bacteria in the gut (14). Therefore, fostering gut microbiota may shed light on improving body weight and restoring health in undernourished children. Moreover, consuming targeted foods to facilitate the domination of beneficial microbes in the gut has been recommended (17).

However, few studies focus on finding whether soy protein can be used as a substitute for casein and whether the alleviation of malnutrition is interrelated with the regulation of intestinal flora. In this study, a comparison between casein and soy protein was performed in a malnourished rat model to shed light on whether soy protein could be substituted in a weaning-phase diet. The present study aimed to (i) compare the effects in an undernourished rat model using soy protein and casein, (ii) measure the contents of SCFAs and metabolites in the serum and colon, (iii) evaluate the change in the structure and relative abundance of gut microbiota using 16S rRNA gene sequencing, (iv) analyze the relationships among microbiota, apparent indicators, and metabolites (in serum and the colon).

## Materials and Methods

### Materials and Chemicals

Normally ground soy protein (>95.2%) powder and casein (>95.2%) were obtained from Gushen Biological Technology Group Co., Ltd. (Dezhou, China). Insulin-like growth factor-1 (IGF-1)ELISA kits were commercially obtained from Abcam (Cambridge, MA, USA). Angiotensin-converting enzyme-2 (ACE 2) ELISA kits were purchased from Biorbyt (Princeton, New Jersey, USA). Acetate, propionate, butyrate, and standard valeric acid were bought from Sigma, Inc. (St. Louis, MO, USA). An E.N.Z.A.® Stool DNA isolation kit that was used to extract DNA was produced by Omega Bio-Tek, Inc. (Norcross, Georgia, USA). All other chemical reagents were of AR grade.

### Analysis of the Nutritional Value of Raw Materials Based on Amino Acids Score

Amino acids score (AAS) was determined as below:


$$\begin{array}{l} \text{AAS\%}=\frac{\left(\text{\%}\right)\text{ sample essential amino acid content}}{\;\left(\text{\%}\right)\text{ recommended essential amino acids}} \end{array}$$

The amino acids of the diet were evaluated using a Hitachi Limited Amino automatic analyzer (L-8900, Tokyo, Japan).

### The Design of Animals and Experiment

Forty male Sprague-Dawley rats (SYXK (Beijing) 2018-0022) (4 weeks from birth, weighing 90.8 ± 4.24 g) were obtained from the Beijing Rital River Laboratory Animal Technology Company (Beijing, China). The malnutrition models were constructed according to the Brown method (18). Briefly, after 7 days of adaptation, 10 randomly distributed rats were categorized as a control group (CTG), and the other 30 rats categorized as a low protein group (LPG) were given a 7% protein diet. After 3 weeks, 30 rats in the malnutrition group (weight was decreased by 51.2% compared to the control rats) were randomly distributed into 3 groups (*n* = 10) as follows: LPG group, casein group (CG), and soy protein group (SPG). The body weight and tail length gain of the rats were measured every week. The experimental diets for each group are listed in Table 1 (Jiangsu Xietong Pharmaceutical Bio-engineering Co., Ltd. Nanjing, China). During the experimental period, water and food were easily and adequately available for the rats. All rats were housed individually in cages placed at the room where temperature (22.3 ± 1°C) and humidity (50.2 ± 10%) were maintained stably with a 12 h light/dark cycle. On day 57 (the body weight had statistically increased in the SPG and CG compared to the LPG), each rat was sacrificed under anesthesia after 12 h of fasting, and blood was immediately obtained to prepare the serum stored at −80°C until analysis. The content of IGF-1 and ACE 2 in the serum were measured according to the instruction of the ELISA kits. The experiment was conducted in accordance with the European Community Guidelines for the Use of Experimental Animals and authorized by the Pony Testing International Group on Animal and Use.

**Table 1:** The composition of diets.

**Component**	**CTG**		**LPG**		**CG**		**SPG**	
	**gm**	**kacl**	**gm**	**kacl**	**gm**	**kacl**	**gm**	**kacl**
Casein/g	200	800	70	280	200	800	-	0
L-Cystine/g	3	12	3	12	3	12	3	12
Soy protein/g	-	-	-	-	-	-	245.4	982.2
Corn Starch/g	397.5	1,590	527.5	2,110	397.5	1,590	352.0	1,407.8
Maltodextrin 10/g	132	528	132	528	132	528	132	528
Sucrose/g	100	400	100	400	100	400	100	400
Cellulose, BW200/g	50	0	50	0	50	0	50	0
Lard/g	70	630	70	630	70	630	70	630
Vitamin Mix V10037/g	10	40	10	40	10	40	10	40
Mineral Mix S10022G/g	35	0	35	0	35	0	35	0
Choline Bitartrate/g	2.5	0	2.5	0	2.5	0	2.5	0
FD&C Red Dye #40/g	-	0	-	0	-	0	0.10	0
Total/g	1,000	4,000	1,000	4,000	1,000	4,000	1,000	4,000
Protein (%)	20.3	20.3	7.30	7.30	20.3	20.3	20.3	20.3
Carbohydrate (%)	64.0	64.0	77.0	77.0	70	64.0	64.0	64.0
Fat (%)	7.00	15.7	7.00	15.7	7.00	15.7	7.00	15.7
Total		100		100		100		100

*CTG, Control group; LPG, low protein group; CG, casein group; SPG, soy protein group*.

### SCFAs Analysis

The concentration of SCFAs was measured as described in a previous study (19) according to Shanghai Biotree Biotech Co., Ltd. (Shanghai, China) protocol. Fifty milligrams of colon contents were extracted with 1 mL ice-cold physiological saline, homogenized in a ball mill (4 min, 40 Hz) (Shanghaijingxin Experimental Technology, Shanghai, China), and then ultrasonically processed for 5 min in ice water. The supernatant obtained by centrifugation (5,000 rpm, 20 min, 4°C) was collected, and added to the mixture (9:1 v/v) containing 25% (w/v) of metaphosphoric acid. After centrifugation, the supernatant was transferred into a fresh vial for analysis. The supernatant (1 μL) was analyzed by an HP-FFAP capillary column (Agilent, Folsom, CA, USA). Using helium as the carrier gas, the initial temperature was set to 80°C (1 min), and subsequently increased to 200°C (in 5 min), then maintained (1 min) at 240°C. The injection and detector temperatures were 250 and 270°C, respectively. The electron impact energy was −70 eV. The mode of MS was set in Scan/SIM mode.

### Untargeted Metabolomics

Sample of colon content (50 mg) was diluted in 1 mL of the extract solution (methanol: acetonitrile: water = 2:2:1, with isotopically-labeled internal standard mixture). After homogenization (35 HZ, 4 min) and sonication (5 min) in three times of repeated ice bath, the samples were incubated for 1 h (−40°C) and finally centrifuged (12,000 g, 15 min, 4°C). The supernatant was transferred to a fresh glass vial for analysis.

One hundred microliters of serum sample were mixed with 400 μL of solution (acetonitrile: methanol = 1:1) and vortexed (30 s), then sonicated (10 min, ice water bath), and subsequently incubated (1 h, −40°C) to precipitate proteins. After centrifugation (4°C, 12,000 rpm, 15 min), the supernatant was ready for analysis.

The quality control sample was prepared by mixing up all the samples with an equal aliquot. Liquid-chromatography tandem mass spectrometry (LC-MS/MS) analysis was conducted by a UHPLC system (Thermo Fisher, Waltham, MA, USA) equipped with BEH amide column (2.1 × 100 mm, 1.7 μm) connected to a Q Exactive HFX mass spectrometer (Thermo Fisher, Waltham, MA, USA). The flow was blended with 25 mmol/L of ammonium acetate and 25 mmol/L of ammonia hydroxide in water (pH = 9.7) (A) and acetonitrile (B). The injected sample was set at the temperature of 4°C, and the sample volume was set at 2 μL. An acquisition software consistently monitored the full scan MS spectrum. The electrospray ionization (ESI) source conditions were coded as follows: sheath gas flow rate and aux gas flow were 30 Arb and 25 Arb, respectively. Temperature was set at 350°C, and full MS resolution and MS/MS resolution were of 60,000 and 7,500. Collision energy was obtained with 10/30/60 in N-channel enhancement (NCE) mode, and spray voltage was found to be either 3.6 kV (positive) or −3.2 kV (negative).

### 16S rRNA Gene Sequences

On day 57, after sacrificing under anesthesia, the colon contents of the rats were collected from the respective groups. The analysis of 16S rRNA gene sequences was performed in the laboratory as per previous publication (19). After extracting total DNA by an E.N.Z.A.® Stool DNA isolation kit (Omega Bio-Tek, Inc. St. Louis, MO, USA), primers were programed according to the conserved regions, and subsequently, sequencing adapters were added to the ends of the primers (forward 5′-ACTCCTACGGGAGGCAGCA-3′ and reverse 5′-GGACTACHVGGGTWTCTAAT-3′). PCR amplification was performed, and the productions were cleaned, quantified, and homogenized to form a sequencing library. The built library was qualified by a library quality inspection and sequenced with an Illumina HiSeq 2500 system (Illumina In., CA, USA). The original image data obtained by Illumina HiSeq platforms were translated into sequenced reads, and stored in FASTQ (referred to as fq) file format. Trimmomatic v0.33 software was first used to filter the raw reads obtained by sequencing; then, cutadapt 1.9.1 software was used to distinguish and cut the primer sequences. FLASH v1.2.7 software was applied to pass the overlapped splices of the high-quality reads of each sample, and clean reads were obtained from the resulting spliced sequence. The UCHIME v 4.2 software was employed to distinguish and cut the chimera sequence to produce the final effective data.

### Statistical Analysis

Data were processed and finally expressed as the mean ± SD. GraphPad Prism 9 Software (San Diego, CA, USA) was used to analyze the differences among the four groups and transformed the data to a figure. For microbiota analyses, the Shannon index evaluated significant differences in the level of alpha diversity among the four groups. The abundance of operational taxonomic unit (OTU) was conducted to compare the changes of diversity among the four groups. The data of the gut microbiota in the levels of genus, host apparent indicators, and metabolites (in colon and serum) were analyzed to evaluate the correlations by Spearman's algorithm. The difference was considered significant if the *p* < 0.05.

## Results

### Amino Acid Composition of Soy Protein and Casein

The amino acid profiles of different proteins are presented in Table 2. A total of 18 amino acids were detected, including 9 essential amino acids. The sulfur amino acids (methionine and cystine) and proline contents in casein reached 4.68 and 10.2%, respectively, while the corresponding values in soy protein were only 2.49 and 5.4%, respectively. However, compared with casein, soy protein was rich in arginine, alanine, glycine, and aspartic acid, and the total amount exceeded 86.8%. As shown in Table 3, the AAS of casein was above 100, which meets the requirements of FAO recommendations. Sulfur-containing amino acids were obviously the limiting amino acids in soy, which had the lowest percentage of all amino acids (2.49%). Tyrosine and phenylalanine, in soy protein and casein, had the highest AAS, reaching the values of 130.5 and 172.9, respectively. The following were the only two amino acids in soy with an AAS below 90: sulfur-containing amino acids (71.5) and lysine (87.4). The AAS is an important index for evaluating the quality of protein; it has been widely used to predict the potential ability of protein in food to provide indispensable amino acids.

**Table 2:** Amino acid composition (mg·g^−1^ protein).

**Amino acid**	**Soy protein**	**Casein**
Tryptophan	7.94 ± 0.30^b^	10.2 ± 0.27^a^
Aspartic acid	89.4 ± 1.51^a^	60.0 ± 2.05^b^
Threonine	29.2 ± 0.31^b^	34.2 ± 0.13^a^
Serine	37.0 ± 2.26^b^	41.5 ± 1.63^a^
Glutamate	147.9 ± 2.33^b^	182.9 ± 2.40^a^
Proline	41.6 ± 0.71^b^	91.8 ± 1.77^a^
Glycine	32.1 ± 2.19^b^	15.7 ± 2.55^a^
Alanine	33.4 ± 1.13^a^	25.7 ± 1.08^b^
Cystine	8.84 ± 0.24^b^	16.5 ± 0.57^a^
Methionine	10.5 ± 0.34^b^	25.5 ± 0.25^a^
Valine	39.1 ± 0.41^b^	57.0 ± 0.34^a^
Isoleucine	38.2 ± 0.37^b^	45.7 ± 0.35^a^
Leucine	62.0 ± 0.34^b^	78.0 ± 0.34^a^
Tyrosine	25.2 ± 0.33^b^	45.1 ± 0.43^a^
Phenylalanine	43.2 ± 0.43^a^	44.8 ± 0.36^a^
Lysine	49.8 ± 0.62^b^	69.0 ± 0.32^a^
Histidine	20.6 ± 0.43^a^	25.0 ± 0.37^a^
Arginine	58.1 ± 2.19^a^	31.1 ± 2.05^b^

*Data are presented as the mean ± SD (n = 3). The letters after the data indicate the statistical difference (p < 0.05)*.

**Table 3:** The amino acid score (AAS) of dietary ingredients.

**AAS (for infants/preschool children 2 yrs)**	**FAO (2013)**	**Soy protein**	**Casein**
Threonine	31	94.2 ± 1.00^b^	110.2 ± 5.59a
Methionine+Cystine	27	71.5 ± 2.13^b^	155.8 ± 2.97^a^
Isoleucine	32	119.2 ± 1.15^b^	142.7 ± 1.08^a^
Leucine	66	93.9 ± 0.51^b^	118.1 ± 0.52^a^
Phenylalanine+Tyrosine	52	130.5 ± 0.62^b^	172.9 ± 1.51^a^
Lysine	57	87.4 ± 1.09^b^	121.0 ± 0.56^a^
Histidine	20	103.1 ± 2.13^b^	124.8 ± 1.86^a^
Tryptophan	9	93.4 ± 3.47^b^	120.3 ± 3.13^a^
Valine	43	91.0 ± 0.96^b^	132.5 ± 0.78^a^

*Data are presented as the mean ± SD (n = 3). The letters after the data indicate the statistical difference (p < 0.05)*.

### Effects of Soy Protein on Body Weight, Tail Length, IGF-1, and ACE 2

**Figure 1:**
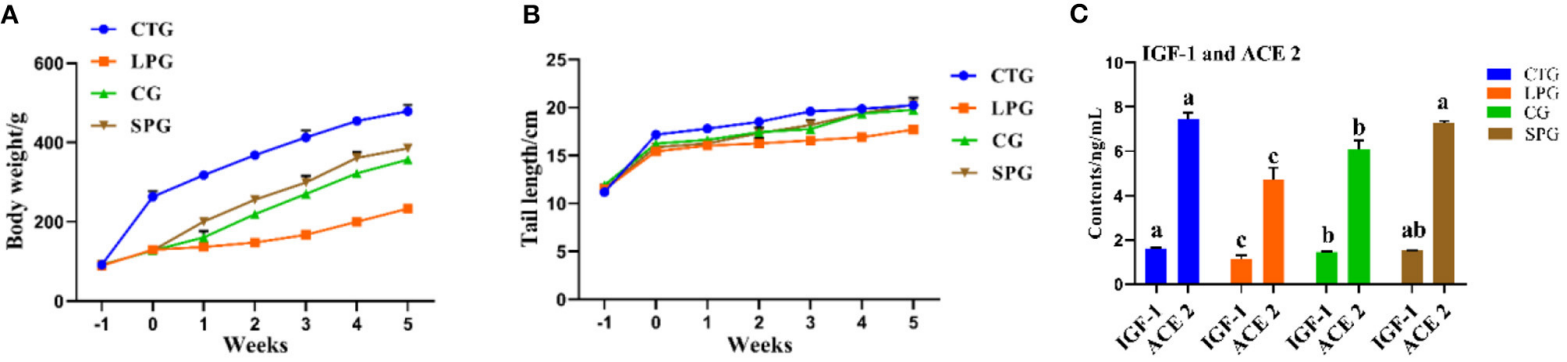
Diet intervention alleviated the physiological condition of rats. **(A)** The body weight of rats. **(B)** The tail length of rats. **(C)** IGF-1 and ACE 2 contents in the serum. Data are presented as mean ± SEM. Different letters indicate statistical differences among the groups (*p* < 0.05). IGF-1, insulin-like growth factor-1; ACE 2, angiotensin-converting enzyme-2; CTG, control group; LPG, low protein group; CG, casein group; SPG, soy protein group.

The undernourished model induced by the protein-deficient diet (7% protein diet) showed a stunted growth in rats, as they reduced an average of 51.2% in body weight after 3 weeks, compared to CTG (Figure 1A). After 6 weeks of supplementary diets, the rats from the SPG and the CG significantly exhibited higher body weight gain (*p* < 0.05) than LPG, which increased by 64.9% in the SPG compared to those in the CG. At the same time, malnourished rats also had corresponding shorter tails. Results showed a similar growth trend performed in the length of the rat tail, with rats in the SPG and CG growing faster than rats in the LPG, and showed no statistical difference with CTG (Figure 1B).

Compared with those in the CTG, rats fed with low protein diet had lower IGF-1 and ACE 2 levels in the serum (Figure 1C). After the administration of supplementary diets (soy protein or casein protein) for 6 weeks, the IGF-1 levels in SPG and CG were elevated by 53.1 and 46.3%, respectively, compared to the LPG (*p* < 0.05). Additionally, the level of ACE 2 increased by 43.0 and 53.1% in the SPG and CG, respectively, compared to the LPG (*p* < 0.05) (Figure 1C).

### Effects of Soy Protein on SCFAs and Colonic Metabolites

**Figure 2:**
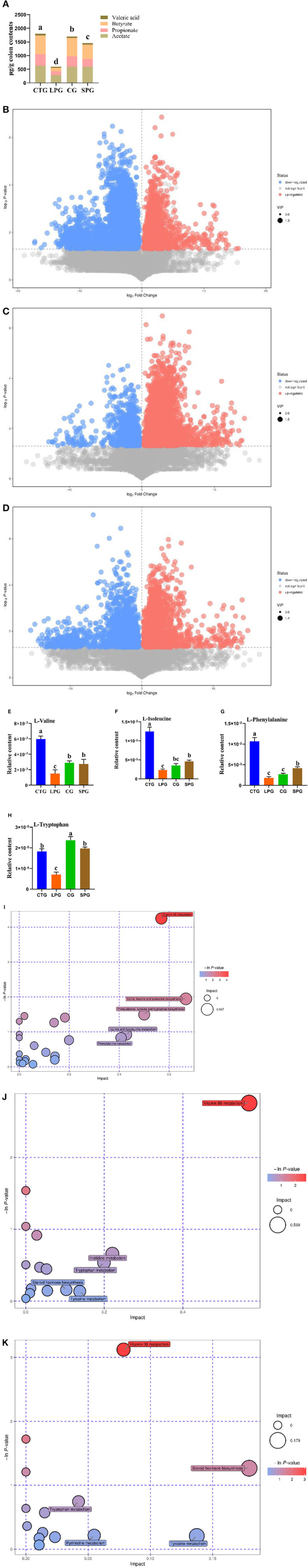
Effect of diet intervention on the metabolites in the colon. **(A)** The contents of short-chain fatty acids (SCFAs). **(B)** The differentially expressed metabolites between LPG and CTG. **(C)** The differentially expressed metabolites between CG and LPG. **(D)** The differentially expressed metabolites between SPG and LPG. **(E)** The relative content of L-valine. **(F)** The relative content of L-isoleucine. **(G)** The relative content of L-phenylalanine. **(H)** The relative content of L-tryptophan. **(I)** Bobble plot of LPG to CTG. **(J)** The bobble plot of CG to LPG. **(K)** The bobble plot of SPG to LPG. Data are presented as mean ± SEM. Different letters indicate statistical differences among groups (*p* < 0.05). CTG, control group; LPG, low protein group; CG, casein group; SPG, soy protein group.

The concentration of total SCFAs and the acetate, propionate, butyrate, and valeric acid in the four groups varied significantly (Figure 2A). The LPG showed a dramatic decrease in the total level of SCFAs by 2.04-fold compared to CTG, while the SPG and the CG showed 1.37- and 1.86-fold increases compared to LPG (Figure 2A). In LPG, the levels of acetate, propionate, butyrate, and valeric acid decreased by 1.19, 1.96, 4.68, and 0.50-fold, respectively, compared to CTG (Figure 2A). Obviously, butyrate production was the most reduced in the LPG, while it was increased by 3.26- and 4.50-fold in the SPG and CG, respectively, compared to the LPG. Moreover, the levels of other SCFAs simultaneously increased after the administration of soy protein in the SPG.

A total of 1,054 metabolites were measured among the four groups. After analysis, 398 differentially expressed metabolites showed significant changes between CTG and LPG (VIP > 1, *p* < 0.05) (Figure 2B), 257 differentially expressed metabolites between CG and LPG (VIP > 1, *p* < 0.05) (Figure 2C), and 292 differentially expressed metabolites between SPG and LPG (VIP > 1, *p* < 0.05) (Figure 2D). In these differentially expressed metabolites, valine was sharply decreased in the LPG by 74.7% compared to the CTG; however, it was restored to 80.6 and 91.2% in SPG and CG, respectively, compared to LPG (Figure 2E). The isoleucine, phenylalanine, and tryptophan also decreased by 81.1, 82.9, and 61.3%, respectively, in the LPG compared to that in CTG (Figures 2F–H), while alleviated after supplementary soy protein and casein.

The Kyoto Encyclopedia of Genes and Genomes (KEGG) was used to catch and map the metabolites in the colonic contents, and the results were analyzed through the enrichment pathway to show the enrichment of metabolites in the pathway. As shown in Figure 2I, the differentially expressed metabolites between CTG and LPG hit 23 metabolism pathways which included vitamin B_6_ metabolism, valine, leucine, and isoleucine biosynthesis, phenylalanine, tyrosine, and tryptophan biosynthesis, pantothenate and CoA biosynthesis, cysteine and methionine metabolism, linoleic acid metabolism, and arginine and proline metabolism [-ln(*p*) > 1]. Similarly, vitamin B_6_ metabolism was significantly influenced in the SPG and CG compared to the LPG [-ln(*p*) > 1] (Figures 2J,K). In addition, amino acid metabolism (tryptophan, tyrosine) and biosynthesis (tryptophan, tyrosine, isoleucine, leucine, valine, and phenylalanine) pathways were statistically changed among CTG, SPG, and CG compared to LPG.

### Effects of Soy Protein on Metabolites in Serum

**Figure 3:**
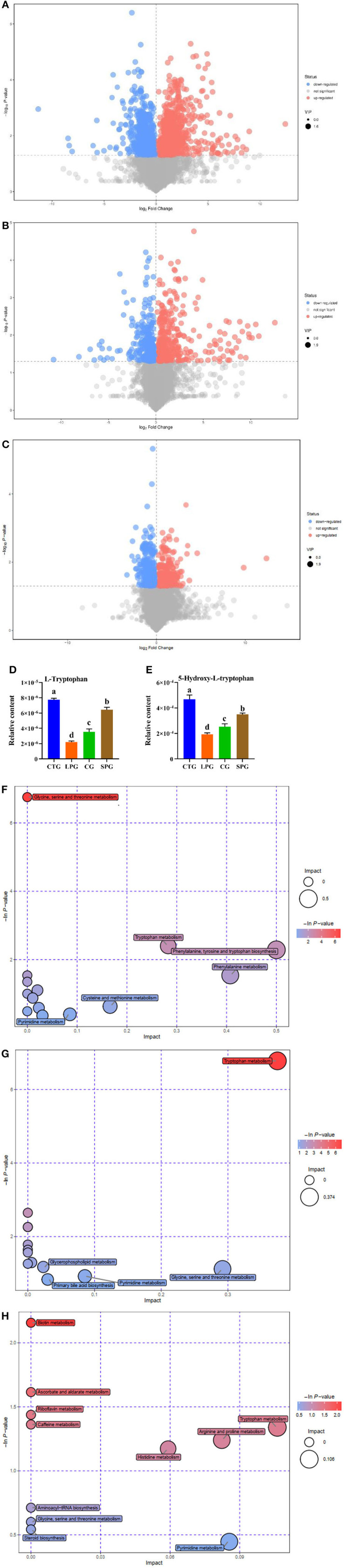
Effect of diet intervention on the metabolites in serum. **(A)** The differentially expressed metabolites between LPG and CTG. **(B)** The differentially expressed metabolites between SPG and CTG. **(C)** The differentially expressed metabolites between CG and CTG. **(D)** The relative content of L-tryptophan. **(E)** The relative content of 5-hydroxy-L-tryptophan. **(F)** Bobble plot of LPG to CTG. **(G)** The bobble plot of SPG to CTG. **(H)** The bobble plot of CG to CTG. Data are presented as mean ± SEM. Different letters indicate statistical differences among the groups (*p* < 0.05). CTG, control group; LPG, low protein group; CG, casein group; SPG, soy protein group.

The metabolites in the serum measured by untargeted metabolomics based on MS/MS data are presented in Figures 3A–C. The LPG had 78 upregulated metabolites and 44 downregulated metabolites compared to CTG (Figure 3A), and the downregulated metabolites were mainly centralized in amino acid substances, such as tryptophan and threonine. However, the number of differentially expressed metabolites was reduced to 81 and 60 in the SPG and CG, respectively, compared to the CTG (Figures 3B,C).

The amount of tryptophan decreased by 266.1% in the LPG compared to CTG while restored in SPG and CG (Figure 3D) (VIP > 1 and *p* ≤ 0.05). Moreover, 5-hydroxy-tryptophan (5-HTP) showed significant changes in the results. In LPG, the content of 5-HTP was sharply decreased by 143.2, 82.2, and 31.5%, respectively, compared to CTG, SPG, and CG, respectively (Figure 3E). Since differences in amino acid substances among groups were observed, we conducted metabolic pathway analysis of the different metabolites (Figures 3F–H). Compared with the CTG, there were 6, 5, and 11 metabolite changes in the pathways of LPG, CG, and SPG, respectively, in which the amino acid metabolism pathway was involved as the main metabolite pathway. Through comprehensive analysis of the pathways where differential metabolites mainly gathered, key pathways that had higher correlation with the metabolites were determined. Five and three differentially expressed metabolites between the CTG and LPG hit the glycine-serine-threonine metabolism pathway and the tryptophan-metabolism pathway. However, these two metabolic pathways were restored among the CTG and the other two groups.

### Effects of Soy Protein on Gut Microbiota

**Figure 4:**
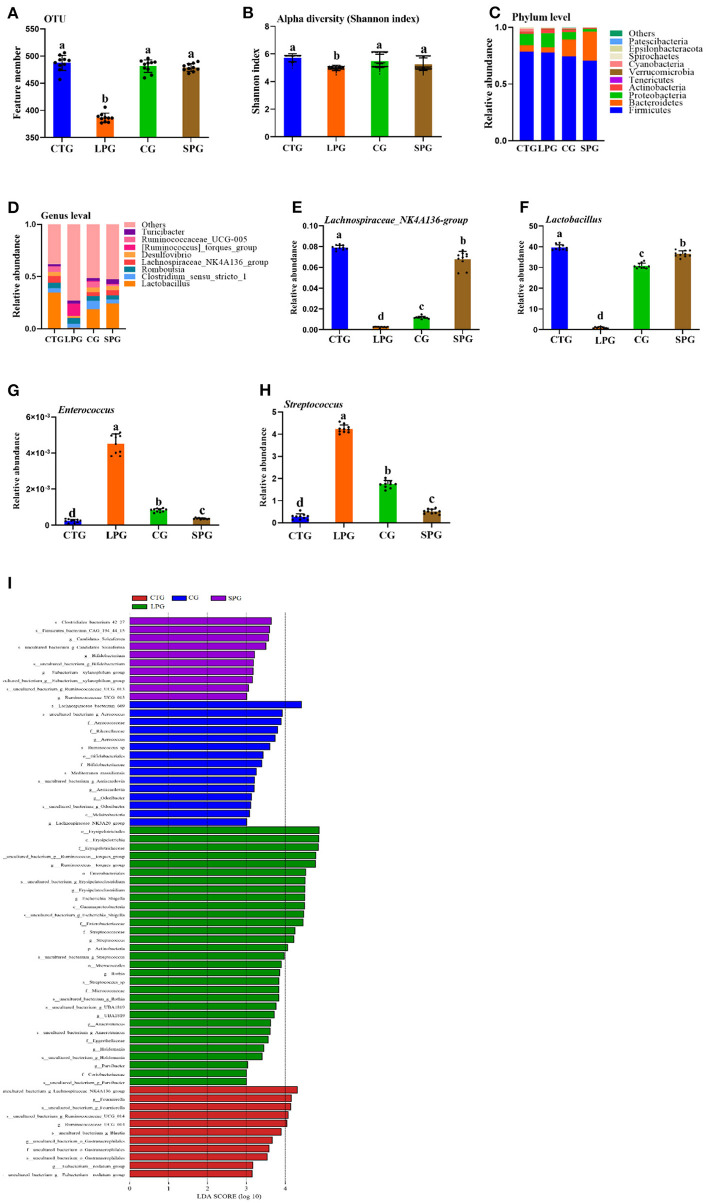
Effect of diet intervention on the gut microbiota. **(A)** The amount of OTU based on 97.0% similarity. **(B)** Shannon index. **(C)** The change in the phylum level. **(D)** The change in the genus level. **(E)** The relative abundance of *Lachnospiraceae_NK4A136- group*. **(F)** The relative abundance of *Lactobacillus*. **(G)** The relative abundance of *Enterococcus*. **(H)** The relative abundance of *Streptococcus*. **(I)** LDA score of four diets on microbiota. Data are presented as mean ± SEM. Different letters indicate statistical differences among the groups (*p* < 0.05). OTU, operational taxonomic unit; CTG, control group; LPG, low protein group; CG, casein group; SPG, soy protein group.

After sampling the colonic contents and sequencing in the V3–V4 regions using 16S rRNA, a total of 2,158,844 pairs of reads were obtained. With double-ended read quality control and splicing, a total of 2,148,662 purified reads were generated. At least 79,107 clean reads were obtained in each sample. Usearch software was used to cluster reads in the levels of 97.0% similarity (20), and subsequently obtained 487, 386, 479, and 481 OTUs in the CTG, LPG, SPG, and CG, respectively (Figure 4A). The significant reduction of OTUs in the LPG suggested that malnutrition caused a sharp decrease in the diversity of bacteria. Similarly, it reflected the richness of microbial communities, and the Shannon index was decreased dramatically in LPG compared to CTG (Figure 4B). However, the OTUs and the Shannon index were upgraded after the rats were supplemented with soy protein and casein, which suggested that the diversity of the species was restored (Figures 2A,B).

The changes in microbiota were shown in both the phylum and genus levels (Figures 4C,D). Belonging to the phylum level, *Firmicutes* dominated among the four groups. However, the ratio of *Bacteroidetes* varied significantly in different groups. *Bacteroidetes* increased significantly after the supplementation of soy protein and casein (Figure 4C). At the genus level, the groups, *Lactobacillus, Clostridium_*sensu_stricto_1, *Romboutsia, Lachnospiraceae_*NK4A136, *Desulfovibrio, Ruminococcaceae_*UCG-005, *[Ruminococcus]_*torques, and *Turicibacter* dominated in the CTG, SPG, and CG, respectively (Figure 4D). However, this trend was reversed in the LPG, with a sharp decrease in the counts of *Lactobacillus* and *Lachnospiraceae_*NK4A136_group and a dramatic rise in the relative abundance of *[Ruminococcus]*_torques_group. Based on the present study and previous conclusions by other scientists, four bacteria were selected for in-depth analysis. The relative abundance of *Lachnospiraceae_*NK4A136_group and *Lactobacillus* increased by 31.0-fold, 3.81-fold and by 3.52-fold, 27.0-fold in the SPG and CG, respectively (Figures 4E,F), while a reverse trend was observed in *Enterococcus* and *Streptococcus* compared to LPG (Figures 4G,H). Compared to LPG, *Enterococcus* and *Streptococcus* decreased by 11.0- and 4.52-fold in the SPG, and simultaneously, it was downregulated by 7- and 1.4-fold in the CG.

A linear discriminant analysis (LDA) column graph apparently showed a differential microbial among the four groups (Figure 4I). Sixty-seven OTUs were observed with significant alterations, including 11 in the CTG, 15 n the CG, and 10 in the SPG, respectively. Importantly, the LPG showed the most notable changes in the number of OTUs (31 OTUs). There were 5 OTUs, 15 OTUs, and 1 OTU in the CTG, LPG and CG, respectively, with LDA scores over 4. As a biomarker among groups, these OTUs were classified at the phylum level, and bacteria found in the LPG were assigned to four OTUs at the phylum level as follows: *Firmicutes, Bacteroidetes, Proteobacteria*, and *Actinobacteria*. Two OTUs were found in the CTG, 4 were found in the CG, and 2 were found in the SPG. However, *Firmicutes* dominated the biomarker bacteria among the four groups.

### The Association Between Microbiota and Apparent Indicators

**Figure 5:**
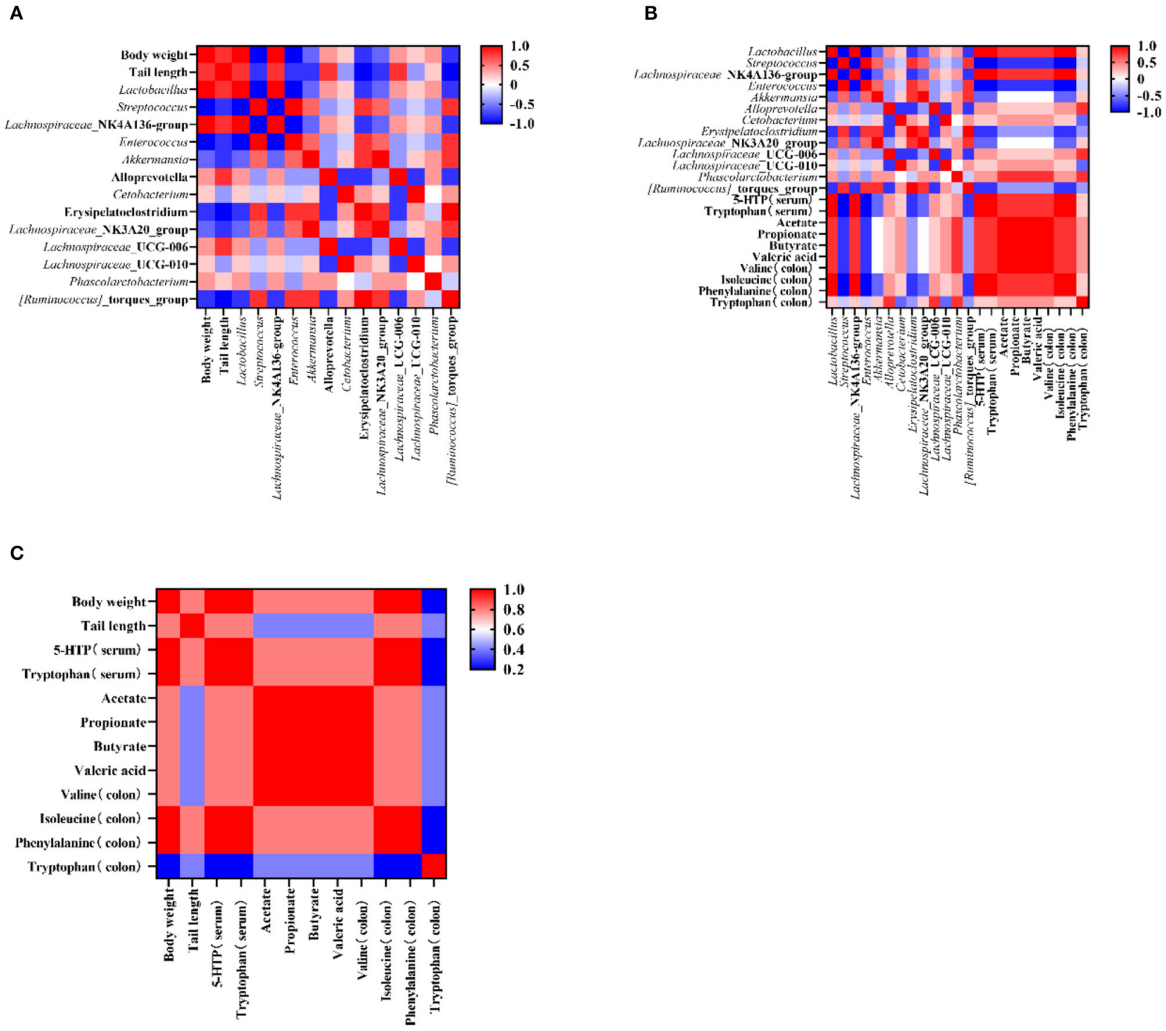
The corelation among the targeted bacteria, apparent indicators, and metabolites (in the serum and the colon). **(A)** The corelation between targeted bacteria and apparent indicators. **(B)** The correlation between targeted bacteria and metabolites (in the colon and the serum). **(C)** The correlation between apparent indicators and metabolites (in the colon and the serum). Data are presented as mean ± SEM. Different letters indicate statistical differences among the groups (*p* < 0.05). CTG, control group; LPG, low protein group; CG, casein group; SPG, soy protein group.

To test the influence of microbiota on rat growth and SCFA production, the Spearman's algorithm was used to carry out a correlation analysis. It was shown that there was a positive correlation among the targeted microbiota (*Lachnospiraceae*_NK4A136_group, *Phascolarctobacterium, Lactobacillus*, and *Alloprevotella*), body weight, and tail length (Figure 5A), while *Enterococcus, Streptococcus, Lachnospiraceae*_NK3A20_group, *Akkermansia, Erysipelatoclostridium*, and *Ruminococcus*_torques_group showed a negative correlation with body weight and tail length. In addition, the change of gut microbiota performed relationships with metabolites in the colon and serum. The alleviation of *Lachnospiraceae*_NK4A136_group, *Lactobacillus*, and *Phascolarctobacterium* were simultaneous with the increase of tryptophan (in the colon and serum), isoleucine (in colon), phenylalanine (in colon), valine (in colon), and 5-HTP (in serum) (Figure 5B). Therefore, an analysis was conducted between metabolites (in the colon and serum) and apparent indicators. It was evident that metabolites in the colon and serum were closely related to the growth of the rats in the malnutrition models (Figure 5C).

## Discussion

Many studies have demonstrated the beneficial functions of dietary intervention on undernutrition by restructuring the gut microbiota (4, 14). However, the potential of plant protein on alleviating growth deficits, and the effect played by gut microbiota and metabolites (in the colon and serum), is still unclear, impelling an urgent need to search for the mechanisms of how soy protein improves undernutrition in rats.

In the present study, supplementary soy protein alleviated the weight loss of weaning rats with undernutrition. Previous findings reported that supplementary soy flour in the diet improved body weight loss in undernourished piglets (4), while soy protein was first evidenced as a functional ingredient which performed extinguished effects on malnutrition in the present study. In addition, changes in the tail length of the rats showed a similar trend with the change in the body weight. In poor areas, deficiency of protein intake is one reason for the stunted growth. Therefore, high quality and efficient protein consumption were important to improve the linear growth of children in the developing countries (21). The amino acids of soy protein and casein in the present study are not completely similar to the previous study because of the difference in the purity and source of soy protein, but the ratio of the amino acids remained the same and the methionine and cystine were found to be the limiting amino acids in soy protein (22). A previous study confirmed that supplementary soy protein significantly improved the essential plasma amino acid concentrations in growth stunted rats which is evidenced in the present study (21). Although casein was higher than soy protein in AAS, soy protein improved the stunted growth better than casein.

A 6 week-diet intervention significantly restored the stunted growth induced by malnutrition. IGF-1, an indispensable factor in growth, is produced by the growth hormone secreted in the pituitary gland (23). ACE 2 plays an important role in regulating protein uptake, gut microbial ecology, and innate immunity (24). Our results demonstrated that the levels of IGF-1 and ACE 2 in LPG was statistically decreased in the serum, but the levels were restored after the intake of soy protein and casein. The decrease of IGF-1 and insufficient microbes in the gut happened simultaneously in mice (25). Similarly, the LPG showed low IGF-1 level and abnormal gut microbiota, while the supplementary soy protein improved this phenomenon. Soy protein is rich in amino acid composition and nutritional composition, but limited studies have reported the potential of soy protein in improving the malnutrition. More importantly, it has been observed that compared with protein derived from animals, plant-based crude protein has lower digestibility (26). Therefore, undigested protein residues can enter the colon as a substrate utilized by microbial to improve the intestinal health. A hypothesis was formed regarding whether soy protein reshapes the gut microbiota, thus correcting malnutrition.

As an important metabolite (acetate, propionate, butyrate, and valeric acid) of microorganisms, the production of SCFAs was related to the effects of directed foods on gut microbiota, which applied protein and cellulose in the ingested diet (27). The total concentration and compositions of SCFAs among the four groups varied significantly. The CTG showed the highest concentration of SCFAs, while the LPG had the lowest concentration, which was similar to the previous study (27). Butyrate has been claimed to foster the proliferation of prebiotics in the gut and it played a unique role in reshaping the host metabolism and maintaining the integrity function of the intestine (28). A previous study claimed that the concentration of soy protein influenced the growth performance and the content of butyrate in broiler chicken through to day 42 of age (29). The sharp reduction in butyrate in the LPG was in accordance with the adverse influence in the gut microbiota, while the restoration and production of butyrate in the SPG also protected gut integrity. Acetate, propionate, and butyrate were dominant in the total SCFAs, while the butyrate content in the LPG was significantly reduced. This result suggests that dietary intervention caused changes in the contents of microbiota metabolites, and were simultaneous with the changes in the intestinal microbes. Overall, soy protein increased the production of SCFAs and influenced gut microbiota, and subsequently alleviated malnutrition. However, changes of other metabolites in the colon coincided with SCFAs and gut microbiota.

The content of tryptophan decreased sharply in the colon in LPG, which indicated that the tryptophan biosynthesis and metabolism was damaged in malnutrition models. As aryl hydrocarbon receptor ligands, tryptophan catabolites played a unique role in the gut and immune system in the host (30). Therefore, the restoration of this metabolism and the content in the colon were critical in malnutrition improvement. At the same time, phenylalanine, isoleucine, and valine were also decreased in LPG which coincided with our previous results (31). Vitamin B_6_ metabolism in the colon showed significant influence among LPG and other groups because the deficiency of tryptophan decreased the tryptophan-nicotinamide conversion rate, while subsequently influenced the vitamin B_6_ metabolism (32). In addition, the disorder of Vitamin B_6_ and low level of propionate and butyrate showed in LPG at the same time. Meanwhile, as a biomarker, *Lachnospiraceae*_NK4A136_group was observed to be a vitamin B_6_ deficient group (33). Therefore, it is concluded that the change of metabolites in the colon was closely associated with the gut microbiota in alleviating undernutrition. Recent studies reported that changes of metabolites in the serum was related to the gut microbiota and SCFAs, which meant that the metabolites from the gut microbiota passed the gut barrier, and subsequently influenced the serum metabolites (28, 34).

Soy protein intake improved health in malnourished rats by influencing the gut microbiota, especially some signaling bacteria. Accumulating evidence has revealed that tryptophan, 5-HTP, and 5-HT (serotine) are important factors to maintain the intestinal balance (28, 30). Distress, loss of appetite, and other symptoms are usually associated as the signs of malnourishment in patients. As a neurotransmitter, 5-HT has been shown to affect the regulatory mechanisms of mood, appetite, etc., and it is associated with stress, anxiety, and depression (35). The results from untargeted metabolism analysis in the serum showed that the 5-HTP levels decreased sharply in the LPG, but its relative content was restored after diet intervention with soy protein and casein. A previous study reported that different diet proteins induced variable changes of 5-HTP and 5-HT in rats (36). In fact, diet soy protein showed higher tryptophan in the serum and higher 5-HTP in the hypothalamic, the hippocampal, and the cortical regions, compared to diet casein (36). Results were not completely the same with the present study, because the rats administered with soy protein in the present study were malnourished. However, the significant increase of 5-HTP in the serum evidenced a greater potential of soy protein in alleviating malnutrition. The 5-HTP could cross the blood-brain-barrier, and regulate the production of 5-HT. Thus, the reduction of 5-HTP in the serum might reveal the change of 5-HT in the brain. The difference of *Lactobacillus* levels between the CTG and LPG was also related to the change of 5-HT, because Liu et al. reported *Lactobacillus* genera on 5-HT synthesis (28).

The relative contents of tryptophan and the precursor of serotonin were significantly reduced in malnourished children which are consistent with our findings of a significant reduction of tryptophan levels in the LPG (24). Dietary supplementation with soy protein and casein alleviated this situation. Although the levels of serum 5-HTP and tryptophan in the SPG and CG did not reach the levels found in the normal rats, the SPG was still more effective than the CG which reported in a previous study that soy protein produced better 5-HTP and tryptophan in the serum than casein in the healthy rats (36). A recent study demonstrated that gut microbes produce 5-HT, 5-HTP, and indolealdehyde (IAld) *via* numerous other metabolic pathways, such as *Lactobacillus*, which converts tryptophan to IAld (28). Our research demonstrated a closer association between the decrease of gut microbiota and the downregulation of tryptophan metabolism in the LPG, while supplementary soy protein to undernourished rats regulated intestinal diversity and upregulated the tryptophan metabolism. Moreover, the glycine-serine-threonine metabolism pathway was significantly enriched among the four groups. In this pathway, compared to CTG, the LPG had hit the greatest number of differentially expressed metabolites (five), while the SPG and CG each had hit one. These data indicate that malnutrition caused by inefficient protein intake led to the disorder of the glycine-serine-threonine metabolism pathway.

The diversity and richness of the gut microbiota in LPG were statistically decreased which coincided with previous reports which showed that the change in the structure and relative content of gut microbiota was related to malnutrition (4). After supplementation with soy protein, the relative abundance of prebiotics, such as *Lachnospiraceae*_NK4A136_group and *Lactobacillus* in the gut increased, which was similar to a previous study in which Tamura et al. (27) observed changes in various bacteria after the intake of soy protein. Additionally, the relative abundance of *Streptococcus*, which was observed to be high in malnourished children (37), was much higher in the LPG and lower in the rats receiving soy protein. Therefore, the decrease of “age-discriminatory” bacteria indicated that the gut microbiota became more maturer and healthier. Moreover, the increase of the relative abundance of *Lactobacillus* and *Lachnospiraceae*_NK4A136_group led the host healthier because *Lactobacillus* terminated the adverse effects of chronic undernutrition in germ-free mice (25). All the bacteria observed in this study were related to intestinal health and maturation, as evidenced by previous studies (4, 28). Studies claimed that SCFAs performed beneficial effects on gut microbiota (28), and subsequently emerging views have focused on the relationship between gut microbiota and metabolites.

In the results of OMICS, fifty bacteria at the genus level were shown to be statistically correlated with the metabolites in the colon and serum, and found to have alleviated the growth defects; especially, the increase of *Lactobacillales, Alloprevotella*, and *Lachnospiraceae_NK4A136_group* kept up with the upregulation of beneficial substances, such as SCFAs and amino acids in the colon and serum. Many studies have found that SCFAs played crucial roles in the metabolites of the colon, serum, intestinal functions, and in growth development (34, 38). It is evident that the gut microbiota influenced the metabolites in the serum and colon, and subsequently improves host health, such as attenuating undernutrition.

## Conclusion

In the present study, soy protein intake positively influenced the physiological condition of rats by increasing the body weight, tail length, and serum bioinformation compared to undernourished rats. The change in the production of SCFAs revealed a change in the composition of gut microbiota. From the results of 16S rRNA sequencing, the relative abundances of four bacteria in the SPG and CG changed significantly compared to that in the LPG. Untargeted metabolic profiling showed that the metabolites of tryptophan and 5-HTP in malnourished rats changed significantly. The tryptophan metabolism and glycine-serine-threonine metabolism pathways were dramatically damaged in LPG but could be restored by supplementing the diet of soy protein. In the present study, we first reported the potential of soy protein in improving malnutrition evidenced by the changes of gut microbiota, SCFAs, colon, and serum metabolites. In addition, the bacteria of *Lachnospiraceae*_NK4A136_group, *Lactobacillus, Enterococcus*, and *Streptococcus* were closely related to malnutrition. More importantly, serum metabolites (tryptophan and 5-HTP), colon metabolites (SCFAs, valine, phenylalanine, isoleucine, and tryptophan), and metabolite pathways (tryptophan and glycine-serine-threonine in serum and vitamin B_6_ and tryptophan in the colon) were shown for the first time, with a significant change during the period of improving malnutrition in early life. This study evidenced that soy protein supplementary is an effective dietary intervention that alleviates malnutrition. Soy protein, an inexpensive and easily obtained ingredient in the food industry, can be added to a supplemented food to reduce early childhood malnutrition.

## Data Availability Statement

The data presented in the study are deposited in the National Center for Biotechnology Information repository, accession number PRJNA774838.

## Ethics Statement

The animal study was reviewed and approved by Beijing Municipal Science and Technology Commission.

## Author Contributions

ZW and NZ contributed equally to this work. ZW and NZ performed the majority of the experiments and wrote the manuscript. LZ and ZS contributed to the data analysis. BD, GR, and YY designed and supervised the study and checked the final manuscript. All authors contributed to the article and approved the submitted version.

## Funding

This work was supported by the Central Public-interest Scientific Institution Basal Research Fund (Y2020PT30).

## Conflict of Interest

The authors declare that the research was conducted in the absence of any commercial or financial relationships that could be construed as a potential conflict of interest.

## Publisher's Note

All claims expressed in this article are solely those of the authors and do not necessarily represent those of their affiliated organizations, or those of the publisher, the editors and the reviewers. Any product that may be evaluated in this article, or claim that may be made by its manufacturer, is not guaranteed or endorsed by the publisher.

## Abbreviations

CTG: control group
LPG: low protein group
CG: casein group
SPG: soy protein group
IGF-1: insulin-like growth factor-1
ACE 2: angiotensin-converting enzyme-2
SCFAs: short-chain fatty acids
5-HTTP: 5-hydroxytryptophan.
